# Expanding co-payment for methadone maintenance services in Vietnam: the importance of addressing health and socioeconomic inequalities

**DOI:** 10.1186/s12913-017-2405-y

**Published:** 2017-07-12

**Authors:** Bach Xuan Tran, Quyen Le Nguyen, Long Hoang Nguyen, Huong Thu Thi Phan, Huong Thi Le, Tho Dinh Tran, Thuc Thi Minh Vu, Carl A. Latkin

**Affiliations:** 1 Institute for Preventive Medicine and Public Health, Hanoi Medical University, Hanoi, Vietnam; 2 Johns Hopkins Bloomberg School of Public Health, Baltimore, MD USA; 3 Authority of HIV/AIDS Control, Ministry of Health, Hanoi, Vietnam; 4 Institute for Global Health Innovations, Duy Tan University, Da Nang, Vietnam; 5 School of Medicine and Pharmacy, Vietnam National University, Hanoi, Vietnam; 6 Department of Hepatobiliary Surgery, Viet-Duc Hospital, Hanoi, Vietnam; 7 Department of Immunology and Allergy, National Otolaryngology Hospital, Hanoi, Vietnam

**Keywords:** Methadone maintenance, Willingness to pay, Contingent valuations, Integrative models, Vietnam

## Abstract

**Background:**

Ensuring high enrollment while mobilizing resources through co-payment services is critical to the success of the methadone maintenance treatment (MMT) program in Vietnam. This study assessed the willingness of patients to pay (WTP) for different MMT services delivery models and determined its associated factors.

**Methods:**

A facility based survey was conducted among 1016 MMT patients (98.7% male, 42% aged 35 or less, and 67% living with spouse) in five MMT clinics in Hanoi and Nam Dinh province in 2013. Socioeconomic, HIV and health status, history of drug use and rehabilitation, and MMT experience were interviewed. WTP was assessed using contingent valuation method, including a set of double-bounded binary questions and a follow-up open-ended question. Point and interval data models were used to estimate maximum willingness to pay.

**Results:**

95.5% patients were willing to pay for MMT at the monthly mean price of US$ 32 (95%CI = 28–35). Higher WTP was associated with higher level of educational attainment, higher income, male sex, and had high expenses on opiates prior to MMT. Patients who reported having any problem in Pain/ Discomfort, and who did not have outpatient care last year were willing to pay less for MMT than others.

**Conclusion:**

High level of WTP supports the co-payment policies as a strategy to mobilize resources for the MMT program in Vietnam. However, it is necessary to ensure equalities across patient groups by acknowledging socioeconomic status of different settings and providing financial supports for disadvantaged patients with severe health status.

## Background

As the home of more than half of drug use population in the world, Asian countries have been inordinately hard hit by the twin epidemics of HIV and substance use [1]. Since most people who are opioid dependent use heroin, opium, or pharmaceutical opioids, mainly through the injecting route, it becomes the major driver of the spread of HIV epidemics in Asia [2–4]. Injecting drug use (IDU) together with high stigma and discrimination by community and the lack of enabling policies are major social and structural barriers to scaling up comprehensive care and treatment services for patients in large injection-driven HIV epidemics [3]. Over the past decade, there have been substantial efforts by Asian governments to expand the coverage of methadone maintenance treatment (MMT) services. In China, Malaysia, Indonesia, and Vietnam, empirical evidence has demonstrated that MMT has brought about significant changes in health, social, and economic well-being of MMT patients and their families [5–11]. Therefore, MMT has become an essential component in national HIV/AIDS plans in many Asian settings.

Among Asian countries, Vietnam is in a precarious situation given a commitment to provide MMT for 80,000 people who inject drug (PWID). To date, MMT has been provided to more than 46.000 drug users at 251 free-standing MMT clinics and Provincial/District Health Centres in 58/63 provinces of Vietnam [12]. Moreover, it has been offered free-of-charge. However, while a substantial number of drug users is remained to reach the target of this program, these foreign aids are rapidly decreasing in recent years, causing the financial burden for the Vietnam Government [13, 14]. Without the financial aids from foreign donors, subsidies from the Government could only contribute up to 50% of total operational cost of HIV-related services, including MMT program, until 2020 [15]. In this case, Vietnam Ministry of Health has prioritized several resource optimization and mobilization policies to ensure the sustainability of HIV program. For instance, in MMT clinics, several HIV-related services such as Harm reduction programs, HIV testing and counselling and antiretroviral therapy (ART) are integrated in order to reduce the operational cost. In addition, the Vietnam Government requires the involvement of provincial budgets and co-payment by users in all HIV-related services [16]. These policies, so far, have been effective in the short run and contributed to the expansion of the service for over 24,000 MMT patients.

Generally, the government policy encourages local authorities to cover investment costs, including facilities, human resources, and training for MMT clinics; meanwhile, patients are supposed to co-pay the fee that covers the medication. Co-payment for treatment is popular in Vietnam health care system, contributing to over 50% of total health expenditure [17]. In terms of MMT, the current fee that has been applied in such socialized service models is US$0.5 that approximates 50% of the unit cost for MMT [14, 18]. However, the application of a universal fee might impede some patient groups from accessing and adhering to the treatment. In other words, patients with different characteristics have different decisions in co-payment. Literature in Vietnam, Taiwan and the United States found that patients with higher education, being employed, having higher income, receiving treatment at clinics in high level of health system were willing to pay for MMT than other patients [19–22]. Meanwhile, patients with higher age, having health problems or not believing in the effectiveness of treatment had lower amount of willingness to pay for drug rehabilitation [19–21]. Evidently, when the co-payment mechanism is implemented, the differences in the capacity to pay for MMT among patients with different characteristics may raise the socio inequalities as generating the barriers for accessing MMT (e.g. patients having lower socio-economic status are unaffordable to pay the service with high fee). Therefore, understanding factors associated with patients’ willingness-to-pay (WTP) for the service is necessary for developing contextualized policies on co-payment MMT services.

A previous study in Vietnam demonstrated a high level of WTP for MMT by current MMT services users, with the mean amount of WTP being US$ 15.9 per month [20]. However, at the price of 50% of unit cost, there was only a half of target population willing to use the service. Moreover, there were several limitations in the previous analysis. First, its sample includes only HIV positive drug users who were approached at antiretroviral treatment clinics, while the majority of current drug users at MMT clinics in Vietnam are HIV-negative. Those patients with HIV/AIDS were more likely to suffer from catastrophic health expenditure; meanwhile HIV-negative drug users might have more hope to prevent HIV infection that affect their WTP for the services [8, 14, 20, 23, 24]. Second, This prior study was conducted at HIV outpatient clinics instead of MMT clinics across levels of health administration of the Vietnamese health system [25, 26]. Consequently, the implication of previous research findings is limited. Thus, the purpose of this study was to assess the WTP for MMT among MMT patients attending different services delivery models and examine its associated factors with focuses on socio-economic status, health and drug use-related characteristics.

## Methods

### Survey design and sampling

During June to August 2013, a cross-sectional study was conducted in Ha Noi and Nam Dinh province, involving five MMT clinics. We selected the two provinces in consultation with program managers at the Vietnam Authority of HIV/AIDS for a purposive comparison of an experienced setting—Hanoi and a new setting—Nam Dinh Province. There were four clinics located at district health centers, namely Tu Liem, Ha Dong, Long Bien, and Xuan Truong, and one clinic located at Nam Dinh Provincial AIDS Center. Criteria for selecting these clinics included 1) delivering MMT services; 2) representing both urban and rural areas, and 3) covering various levels of health system such as provincial- and district- levels. The characteristics of study sites are listed in Table 1.

**Table 1:** Study settings and sample size

Level	Settings	Site Name	Type of services	Sample size
Province	Nam Dinh City	Provincial AIDS Center (PAC)	MMT+ VCT	270
District (rural)	Xuan Truong District	District Health Center (DHC)	MMT+ VCT + ART + GH	151
District (urban)	Tu Liem District	District Health Center	MMT+ VCT + ART + GH	201
District (urban)	Long Bien District	District Health Center	MMT+ VCT + ART + GH	184
District (urban)	Ha Dong District	Regional Polyclinic (RPC)	MMT+ GH	210

*VCT* Voluntary HIV Counselling and Testing, *ART* Antiretroviral Treatment, *GH* General Healthcare

A convenience sample of 1016 patients was enrolled in the study, accounting for 80–90% of the sample frame. Interviewers were master students in Public Health at Hanoi Medical University who were working in the field of HIV and who were not affiliated with the clinics where they invited patients to participate.

Survey participants comprised clients who met following inclusion criteria: 1) were taking or initiating MMT at the selected sites’ 2) visited the clinics during the; and 3) aged 18 years and above. First, we assessed the selection criteria among patients and then invited eligible patients to a designated counselling room for the interviews. After that, we introduced the survey to patients and asked them to provide written informed consent if they agreed to participate. Finally, we interviewed them with structured questionnaires.

### Measures and instruments

Face-to-face interviews were conducted by well-trained interviewers using structured questionnaires to collect data on socioeconomic characteristics, health status, history of drug use and rehabilitation, and experience with current MMT. Monthly per capita household income was self-reported by patients and included all sources of income for each household member. Household expenditures were estimated on the basis of recurring expenses (e.g. food, utilities, rent and education) in the past month and non-recurring expenses (e.g. construction, health care, furniture, travel and entertainment) in the past year [27, 28]. Equivalent costs of these goods in 2013 were estimated. Health status in five dimensions (mobility, self-care, usual activities, pain/discomfort and anxiety/depression) was measured using the five-level EQ-5D (EQ-5D-5 L) instrument (EuroQol Group, Rotterdam, Netherlands) [29].

### Measurement of willingness to pay

**Fig. 1:**
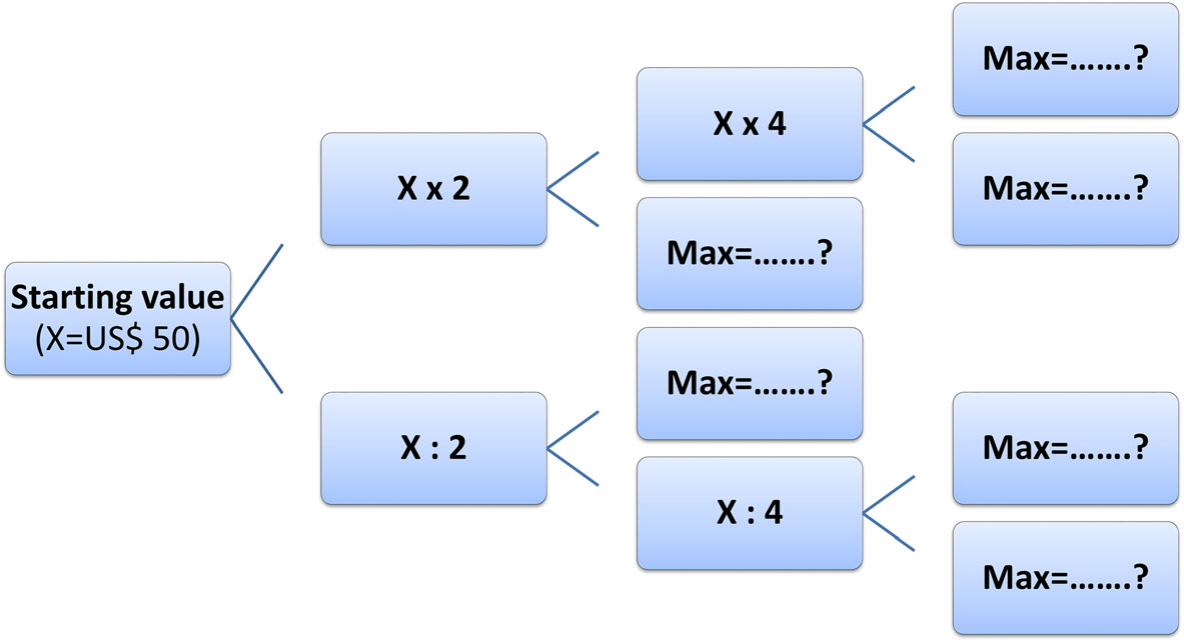
Contingent valuation questions

WTP for MMT was assessed using contingent valuation (CV) method [20], by which all patients were clearly presented “the scenario” that they would value:

- *The problem:* Patients were reminded the negative impacts of opioid use on HIV prevention, care, and treatment. This included an increased risks of transmitting HIV to others if they shared needles and syringes, a sub-optimal adherence and poorer outcomes of HIV/AIDS treatment, and a deteriorated health status and quality of life [30–33]. Also, other socioeconomic impacts of opioid dependence were discussed, for instance, stigma and discrimination, economic burden, and poverty risk of households. Traditional drug rehabilitation services available for opioid users showed limited long-term efficacy, and a large proportion of patients relapsed to drug abuse after several rehabilitation periods.
- *The attributes of MMT services:* The patients were then introduced into the effectiveness of MMT as a substitution therapy for opioid dependants. MMT is cost-effective in reducing the frequency of opioid use, improving health status and quality of life of patients, supporting adherence to ART [9, 34, 35]. In addition, drug users taking MMT can have earlier access to health care services, receive adequate health information, counselling, and referrals, which in turn, may reduce other risky health behaviours, for example, alcohol use disorders [31]. Furthermore, patients can continue to be productive, released from stigma, discrimination, and financial burden of opioid as well as health care expenses associated with opioid abuse [34].
- *The market*: The patients were presented the scale-up plan of MMT services that would be offered to them or their family members. Patients would be required to visit the MMT clinics once a day to take MMT under direct supervisions of health care workers. Currently, MMT services are delivered by the public health care system, and offered free-of-charge, however, its coverage is very low as of 20% by 2013 [36]. Since the international financial support is decreasing, it would be difficult for the Government to expand the coverage of this service. The patients were then asked the maximum they were willing to pay out-of-pocket for the MMT services.
- *The CV method*: has been widely used as a valid method for eliciting patients’ preference and WTP and has been applied in the previous study in Vietnam [20]. Double-bounded dichotomous choice questions backed by an open-ended question were used for eliciting patients’ WTP for MMT services. Initially, patients were asked if they were willing to pay for a monthly fee of 1,000,000 Vietnam Dong (~US$ 50) to take MMT. This starting value was the most-update estimation of unit cost for one-month MMT. If the respondent indicated a WTP the first amount offered, then interviewers asked a follow-up question with the new threshold at double the first one. If the respondent was unwilling to pay, interviewers then halved the price. The question was repeated until the amount to be offered was four times or one-fourth of the starting value. Finally, patients were asked an open-ended question follows the double-bounded binary questions: “What is the maximum price you would be willing to pay per month for the MMT?” Fig. 1. presents the details of CV questions.

### Statistical analysis

Student-t and Chi-square tests were used to examine the differences in opioid use behaviours among MMT patients in different sites. Since WTP was interviewed using both double-bounded and open-ended questions, it included a mixture of censored and uncensored data. The *point and interval data models*, which consist of interval data model and simple Tobit model, were used to estimate the average WTP for MMT services by different patients groups [37].

In the typical interval model, the WTP is supposed to have lognormal distribution, which is calculated by formula as below [37]:

$$ {logWTP}_i^{\ast }={x\prime}_i\beta +{\varepsilon}_i $$

Where WTP_i_ represents the true value of WTP of patient *i*; *x’*_*i*_ denotes a vector of independent variables and *ε*_*i*_ represents a random element (with normal distribution, mean zero and standard deviation σ). In the interval model, the interval censoring means that the value of WTP is between the selected bid (denoted *t*_*li*_) and the next bid in the scale (denoted *t*_*ui*_). Thus, the logWTP* also lies in (log*t*_*li*_; log*t*_*ui*_). The right censoring means that logWTP* is higher than log*t*_*ui*_; and the left censoring refers that logWTP* is lower than log*t*_*li*_ *.* As a result, the log-likelihood function is constituted by three components (interval censoring, right censoring and left censoring) as below [37]:

$$logL=\sum\limits^{N}_{i=1}
\left\{
\begin{array}{ll}
I^a_i\ \text{log}\ \left(\upphi\left(\frac{logt_{ui}-x^{\prime}{~}_{i}\beta}{\sigma}\right)-\upphi\left(\frac{logt_{li}-x^{\prime}{~}_{i}\beta}{\sigma}\right)\right)\\
+I^b_i\ \text{log}\ \left(1-\upphi\left(\frac{logt_{ui}-x^{\prime}{~}_{i}\beta_2}{\sigma}\right)\right)+I^c_i\ \text{log}\ \left(\upphi\left(\frac{logt_{ui}-x^{\prime}{~}_{i}\beta}{\sigma}\right)\right)
\end{array}
\right\}$$

Where *logL* means Log-likelihood function; σ is the scale parameter; ɸ denotes the cumulative standard normal density function; *I* ^*a*^_*i,*_ *I* ^*b*^_*i*_ and *I* ^*c*^_*i*_ are binary variables (value 0/1 options) that have value 1 if the data are treated as (a) interval censored (*I* ^*a*^_*i*_ *= 1*_*,*_ *I* ^*b*^_*i*_ *= 0* and *I* ^*c*^_*i*_ *= 0)*; or (b) right-censored (*I* ^*a*^_*i*_ *= 0*_*,*_ *I* ^*b*^_*i*_ *= 1* and *I* ^*c*^_*i*_ *= 0)*; or (c) left-censored (*I* ^*a*^_*i*_ *= 0*_*,*_ *I* ^*b*^_*i*_ *= 0* and *I* ^*c*^_*i*_ *= 1)* [37].

For example, a patient selected the highest bid in WTP scale (i.e. US$ 200) and he states US$ 220 as a maximum point. In this case, the data are coded as interval censored if we assume WTP to be between *t*_*li*_ = 200 and *t*_*ui*_ = 220. The observation is treated as right-censored if we assume WTP to be more than *t*_*li*_ = 220. Otherwise, if this patient is not willing to pay even the lowest bid in WTP scale (i.e. US$ 12.5), the observation is considered left-censored with WTP being inferior to *t*_*ui*_ = 12.5. Zero responses from patients who are not willing to pay are considered left-censored data [38].

Meanwhile, in the point and interval models, if the patients state the points (below the lowest bid or above the highest bid), these observations are coded as uncensored data; while the others are considered censored data. In this case, the model includes four components: three components (censored data) from the interval data model and one component (uncensored data) from the simple Tobit model:

$$ logL=\sum\limits^{N}_{i=1}
\left\{
\begin{array}{ll}
I^a_i\ \text{log}\ \left(\upphi\left(\frac{logt_{ui}-x^{\prime}{~}_{i}\beta}{\sigma}\right)-\upphi\left(\frac{logt_{li}-x^{\prime}{~}_{i}\beta}{\sigma}\right)\right)\\
+I^b_i\ \text{log}\ \left(1-\upphi\left(\frac{logt_{ui}-x^{\prime}{~}_{i}\beta_2}{\sigma}\right)\right)+I^c_i\ \text{log}\ \left(\upphi\left(\frac{logt_{ui}-x^{\prime}_{i}\beta}{\sigma}\right)\right)\\
+I^{d}_i \left(- log{\sigma}_{i} + log\phi\left(\upphi\left(\frac{logWTP^{*}_{i}-x^{\prime}_{i}\beta}{\sigma}\right)\right)\right)
\end{array}
\right\} $$

Where *φ* denotes the probability density function of the standard normal distribution. When we ask: “What is the maximum price you would be willing to pay per month for the MMT?”, if a patient states his WTP of US$ 220 after selecting the highest bid, this observation is considered uncensored (WTP* = 220, *I* ^*a*^_*i*_ *= 0*_*,*_ *I* ^*b*^_*i*_ *= 0, I* ^*c*^_*i*_ *= 0 and I* ^*d*^_*i*_ *= 1*).

In the point and interval data models, because we know the threshold (*t*_*li*_; *t*_*ui*_), we can estimate the coefficient (β) and the scale parameter (σ). In addition, the marginal effects of independent variables on increasing or decreasing WTP value can also be computed [38].

The mean WTP is calculated by using formula with the intercept of the models:

$$ Mean\  WTP= \exp \left({\beta}_0+\frac{\sigma^2}{2}\right) $$

In multivariable analysis, determinants of patients’ WTP were examined, including an “a priori” defined set of candidate variables: *1) socio-demographics:* sex, age, education, marital status, employment, 2) *economic status:* household’s income and capacity-to-pay, 3) *opioid use behaviours:* current use, experienced drug rehabilitation, length since first opioid use, frequency of opioid use, opioid expenses, 2) *clinical characteristics:* HIV stages, CD4 cell count, length of ART, and currently in MMT 3) *health status:* reported having problems in each EQ-5D dimension. The reduced model was constructed using a stepwise forward selection strategy, which included variables based on the log-likelihood ratio test at a *p*-value <0.1, and excluded variables at *p*-values >0.2 [39].

## Results

Characteristics of respondents in each site are presented in Table 2. The majority the study sample was male (98.7%), about two-thirds (67.5%) were living with spouse or partners; 44.7% completed high school or above, and 53.4% were self-employed. The proportion of MMT patients living with spouse was lowest at Nam Dinh PAC (54.8%), and unemployment was highest at Tu Liem DHC (31.8%). The differences among clinics were found in marital status, education attainment, employment and religion (*p* < 0.05).

**Table 2:** Demographic characteristics of respondents by MMT sites

	Nam Dinh		Xuan Truong		Tu Liem		Long Bien		Ha Dong		Total		*p*-value
	N	%	N	%	N	%	N	%	N	%	N	%	
Age													
18- <30	35	13.0	27	17.9	40	19.9	18	9.8	40	19.1	160	15.8	0.05
30- <35	80	29.6	36	23.8	51	25.4	46	25.0	53	25.2	266	26.2	
35- <40	81	30.0	33	21.9	58	28.9	51	27.7	43	20.5	266	26.2	
40- <45	38	14.1	27	17.9	27	13.4	33	17.9	42	20.0	167	16.4	
> =45	36	13.3	28	18.5	25	12.4	36	19.6	32	15.2	157	15.5	
Sex													
Male	266	98.5	151	100.0	199	99.0	181	98.4	206	98.1	1003	98.7	0.56
Female	4	1.5	0	0.0	2	1.0	3	1.6	4	1.9	13	1.3	
Marital status													
Single	101	37.4	29	19.2	44	21.9	30	16.3	47	22.4	251	24.7	<0.01
Live with spouse	148	54.8	116	76.8	135	67.2	139	75.5	147	70.0	685	67.4	
Live with partner	0	0.0	0	0.0	1	0.5	1	0.5	1	0.5	3	0.3	
Divorced	19	7.0	6	4.0	20	10.0	12	6.5	15	7.1	72	7.1	
Widow	2	0.7	0	0.0	1	0.5	2	1.1	0	0.0	5	0.5	
Educational attainment													
Illiterate	4	1.5	1	0.7	5	2.5	3	1.6	4	1.9	17	1.7	<0.01
Elementary	21	7.8	29	19.2	23	11.4	19	10.3	27	12.9	119	11.7	
Secondary	103	38.2	87	57.6	82	40.8	68	37.0	86	41.0	426	41.9	
High	121	44.8	28	18.5	74	36.8	83	45.1	81	38.6	387	38.1	
Vocational	12	4.4	5	3.3	6	3.0	2	1.1	7	3.3	32	3.2	
University	9	3.3	1	0.7	11	5.5	9	4.9	5	2.4	35	3.4	
Employment													
Unemployed	76	28.2	25	16.6	64	31.8	41	22.3	53	25.2	259	25.5	<0.01
Self-employed	159	58.9	67	44.4	94	46.8	110	59.8	112	53.3	542	53.4	
White collars	5	1.9	1	0.7	6	3.0	5	2.7	5	2.4	22	2.2	
Workers, Farmers	10	3.7	54	35.8	11	5.5	7	3.8	18	8.6	100	9.8	
Students	0	0.0	0	0.0	2	1.0	0	0.0	0	0.0	2	0.2	
Other jobs	20	7.4	4	2.7	24	11.9	21	11.4	22	10.5	91	9.0	
Religion													
Cult of ancestors	247	91.5	96	63.6	184	91.5	171	92.9	198	94.3	896	88.2	<0.01
Buddhism	13	4.8	16	10.6	11	5.5	9	4.9	10	4.8	59	5.8	
Catholic	10	3.7	39	25.8	4	2.0	1	0.5	2	1.0	56	5.5	
Protestant	0	0.0	0	0.0	2	1.0	3	1.6	0	0.0	5	0.5	

In Table 3, we compared drug use history of patient across settings. In general, 8.6% patients were HIV-positive and 73.4% ever injected drug. The types of previous drug rehabilitation were diverse across settings. At Tu Liem, Long Bien and Ha Dong clinics, more than half of respondents had experience with private voluntary centers, and this was higher than at Nam Dinh and Xuan Truong clinics (*p* < 0.05). In Xuan Truong DHC, the rural setting, the proportion of having drug rehabilitation at voluntary and compulsory centers was the lowest as of 36.8% and 9.6%, respectively compared to other clinics (*p* < 0.05). In rural and suburban areas, Xuan Truong DHC and Ha Dong PRC, patients had fewer number of previous drug rehabilitation episodes in comparison with other clinics (*p* < 0.05). On average, patients reported spending 295,000 Vietnamese Dong (15 USD) per day for opiates use.

**Table 3:** History of drug use and treatment among respondents across MMT sites

	Nam Dinh			Xuan Truong			Tu Liem			Long Bien			Ha Dong			Total		*p*-value	
	N	%		N	%		N	%		N	%		N	%		N	%		
HIV positive	22	8.4		7	5.3		25	13.2		19	11.2		9	4.4		82	8.6		0.01
ART	16	5.9		6	4.0		23	11.4		15	8.2		6	2.9		66	6.5		0.02
Ever inject drug	222	82.2		91	60.3		153	76.1		119	64.7		161	76.7		746	73.4		<0.01
Current drug use	15	5.6		17	11.3		0	0.0		6	3.3		11	5.2		49	4.8		<0.01
Location of previous drug rehabilitation																			
Home	213	85.9		103	75.7		122	63.5		104	59.8		132	68.8		674	71.6		<0.01
Private voluntary centre	108	43.6		50	36.8		97	50.5		98	56.3		97	50.5		450	47.8		0.01
Compulsory center	74	29.8		13	9.6		68	35.4		69	39.7		32	16.7		256	27.2		<0.01
# drug rehabilitation																			
None	22	8.2		15	9.9		9	4.5		10	5.4		18	8.6		74	7.3		<0.01
1--5 episodes	155	57.4		110	72.9		138	68.7		121	65.8		151	71.9		675	66.4		
6--10	72	26.7		24	15.9		45	22.4		37	20.1		33	15.7		211	20.8		
> 10	21	7.8		2	1.3		9	4.5		16	8.7		8	3.8		56	5.5		
	Mean	95% CI		Mean	95% CI		Mean	95% CI		Mean	95% CI		Mean	95% CI		Mean	95% CI		
Daily cost of drug use (1000 vnd)	405	318	492	251	200	303	444	175	714	299	172	427	356	245	468	336	295	376	0.02
Duration on MMT (month)	11.5	10.6	12.4	9.3	8.4	10.1	27.7	26.0	29.4	18.7	17.2	20.2	15.2	14.1	16.3	16.3	15.6	17.0	0.02

**Fig. 2:**
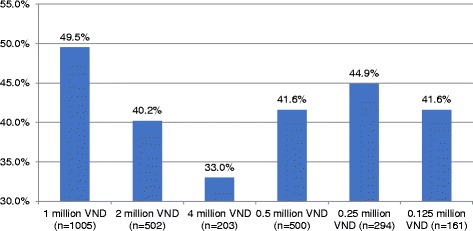
Proportion of participants willing to pay for different bids

Figure 2 reveals the proportion of participants willing to pay for different bids. There was willingness to pay for MMT services data on 95.5% patients. They reported a WTP average price of 639,000 Vietnamese Dong (32 USD) per month. Patients with HIV/AIDS or not-yet-on MMT or never experienced other drug rehabilitation reported lower price of WTP. Patients in Hanoi were willing to pay more for MMT than those in Nam Dinh Province (Table 4).

**Table 4:** Willingness to pay for MMT by different patient groups

Variable		Willing to pay		Monthly price (1000 Vietnam Dong)		
	N	No.	%	Mean	(95% CI)	
Overall	1016	970	95.5%	639	575	704
MMT models						
Nam Dinh	270	259	95.9%	595	478	712
Xuan Truong	151	148	98.0%	594	433	755
Tu Liem	201	193	96.0%	694	539	849
Long Bien	184	174	94.6%	689	532	845
Ha Dong	210	196	93.3%	631	489	774
History of drug rehabilitation						
None	74	69	93.2%	495	298	691
1–5 times	675	648	96.0%	639	559	718
6–10 times	211	202	95.7%	689	542	836
> 10 times	56	51	91.1%	636	369	904
HIV status						
Positive	82	45	54.9%	435	237	632
Negative	876	870	99.3%	660	591	730
N/A	58	55	94.8%	622	325	919
Duration on MMT						
Not yet	28	24	85.7%	545	135	954
1–3 months	115	110	95.7%	815	581	1049
4–6 months	74	72	97.3%	609	370	848
7–12 months	257	246	95.7%	607	483	730
13–24 months	341	327	95.9%	582	482	682
25–36 months	166	157	94.6%	598	450	747
37–60 months	35	34	97.1%	1102	600	1604

In Table 5, we determined factors associated with WTP for MMT with focus on different service delivery models. We found that the WTP of patients taking MMT at urban DHC or suburban RPC was significantly lower than those attending rural DHC or in facility without comprehensive HIV or general health care. Socioeconomic status, health status and drug-related characteristics were found to be predictors of WTP for MMT. Higher WTP was associated with higher level of educational attainment, higher income, male sex, and had high expenses on opiates prior to MMT. HIV status did not remain significant in the reduced model, rather, those patients who reported having any problem in Pain/Discomfort, and who did not have outpatient care last year were willing to pay less for MMT than others. Duration on MMT, number of years of addiction, and times of previous drug rehabilitation episodes were excluded in the reduced model.

**Table 5:** Factors associated with patients’ willingness to pay for MMT services. (Unit: 1000 Vietnam Dong)

	Coef.	95% CI
MMT model (MMT + VCT - ref)		
Rural MMT-ART-VCT-GH (dropped)		
Urban MMT-ART-VCT-GH	−188***	(−262; −113)
MMT + Regional poly clinic	−174***	(−257; −92)
Educational attainment (Illiterate - ref)		
Elementary	128**	(30; 226)
Secondary	75**	(11; 139)
Vocational	200**	(32; 368)
Age groups (18- < 25 - ref)		
40- <45	77*	(−7; 162)
Self-reported health problems (vs. No)		
Usual activities	−92	(−225; 42)
Pain/ Discomfort	−140***	(−222; −57)
HIV negative vs. positive	75	(−22; 171)
Had outpatient care last year (vs. No)	79**	(13; 146)
Historical expenses on opiates (5 levels)		
Highest vs. Lowest	117***	(43; 190)
Sex: female vs. male	−238*	(−515; 40)
Income per capita (Lowest - ref)		
Medium	65	(−18; 148)
High	112***	(32; 192)
Highest	124***	(45; 204)
Constant	253***	(172; 334)

*p < 0.1; **p < 0.05; ***p < 0.01

## Discussion

This study assessed the willingness of drug users to pay for MMT services. Involving a large number of patients in two epicenters of injection-driven HIV epidemics in Vietnam, we were interested to examine if the integration of MMT with other HIV/AIDS or general health care services may influence patients’ WTP. We found that almost all patients were willing to pay for MMT service at an average price of 31 USD per month, approximately the unit cost for providing the service [20]. The availability of other health care services did not increase the WTP of patients. However, those with better physical health status were willing to pay less for MMT. Better socioeconomic status and great level of drug addiction, measured by reported money spent on opiates, significantly predicted higher price that patients were willing to pay for MMT.

To date, this is the largest health facility survey to examine the WTP for MMT services. In the literature, there were few studies in developed countries that showed the willingness of patients to pay for drug rehabilitations [19, 21, 22]. For example, Bishai et al. estimated a WTP a greater monthly amount for drug rehabilitation in Baltimore, Maryland (US$ 29–64) [22]. Zarkin estimated a WTP US$37 for substance abuse treatment among drug users in North Carolina and New York [19]. This is an advancement of previous research in assessing the WTP for MMT services in Vietnam that only involved a small number of HIV positive drug users in HIV outpatient clinics [20]. In the current study, we conducted the survey at various MMT service models including both HIV-negative and HIV-positive patients. Comparing to a prior assessment, respondents in this study sample reported less expenses related to opiates use and more WTP for the MMT services [20]. However, those patients with physical health problems had a lower WTP for MMT similar to patients with HIV/AIDS in the previous study. Findings of this study also support previous work that income, educational attainment, and better health status predicted a lower amount of WTP for MMT [20]. In addition, we further explored that expenses on opiates use previously and having outpatient care predicted higher amount of WTP. It appeared that those patients who had better socioeconomic status or more sufferings from addiction in the past have greater demand for the service than others.

Notably, this study found that patients attending to the MMT clinics with comprehensive care services (comprises MMT, ART, VCT, GH) were willing to pay less amount than those in the MMT clinic with only VCT. The reason for this phenomenon is still unclear and should be elucidated in further studies. However, we assume several explanations based on previous literatures. First, the clinic providing MMT and VCT is located in provincial level, while other clinics are placed in district level. A previous study in Vietnam suggested that the amount of WTP for MMT among patients in provincial clinics were higher than patients in district clinics and even central clinics [20]. Second, patients in the comprehensive clinics had to experience a higher out-of-pocket payment for health services than those in their counterpart, especially patients in the rural clinic [28]. Another analysis in general Vietnamese population from 2002 to 2010 shows that people in rural area were more likely to suffer catastrophic expenditure and impoverishment than those in urban setting [40].

While both availability of comprehensive health care services and duration on MMT did not clearly predict WTP for MMT, implications of this study’s findings mainly focus on individual factors. First, given the high WTP, scaling up co-payment MMT clinics is feasible and can be an effective strategy to mobilize resources to sustain the MMT program as well as the HIV/AIDS system in Vietnam. Second, policies on co-payment MMT services should acknowledge the differences in socioeconomic status of target population; using the average income per capita as a reliable basis for justifying the user fee in each setting. Finally, there should be continuing financial supports for those patients living with HIV/AIDS and who had poor health status. These patient groups are not only economically vulnerable to health care costs but also less motivated to take MMT. It is surprising that those with Pain/ Discomfort were less willing to pay for MMT. Perhaps the MMT was not at a sufficiently high dose or that they didn’t perceive that MMT is adequately helping them. These results suggest the need for pain management for some MMT patients. As it has been known that WTP is associated with medical care retention and compliance, therefore, better case management and support for severe patients is critical, especially in integrative MMT models where patients had complicated health care demand [41]. In a longitudinal study in Vietnam, HIV disease stage and drug interaction between antiretroviral or TB drugs and MMT predict patients’ ongoing drug use during MMT [42] and should be taken into account in any programs that required co-payments.

The strengths of this study include a large sample size of MMT patients in various settings in two epicenters of Vietnam. However, there are several limitations should be acknowledged. First, convenient sampling technique might limit the representativeness of the sample and the capacity to generalize the findings to all MMT patients [43, 44]. In addition, our limitation is that we only selected one clinic at the provincial level; therefore, further research should be warranted in a larger scale with more provincial sites to increase the representativeness of this level of health system. Second, we were not able to collect the MMT dose and cost data. Nonetheless, as the first assessment in different MMT facilities, findings of this study are helpful for developing co-payment policies for MMT services in Vietnam.

## Conclusion

In conclusion, co-payment policies can be applied to MMT services as a strategy to mobilize resources for the program. Also, it is necessary to ensure equalities across patient groups by acknowledging socioeconomic status of different settings and providing financial supports for disadvantaged patients with poor health status. Given the economic vulnerability of drug users, future research may focus on household’s capacity- and willingness-to pay, and interventions to economically empower them.

## Abbreviations

AIDS: Acquired Immune Deficiency Syndrome
**ART**: Antiretroviral therapy
**CV**: Contingent valuation
**DHC**: District Health Center
**EQ-5D-5 L**: EuroQol – 5 dimensions – 5 levels
**GH**: General Healthcare
**HIV**: Human Immunodeficiency Virus
**IDU**: Injecting drug use
**MMT**: Methadone maintenance treatment
**PAC**: Provincial AIDS Center
**PWID**: People who inject drugs
**VAS**: Visual analogue scale
**VCT**: Voluntary counselling and testing services
**WTP**: Willingness to pay
